# Localization and discrimination of GG mismatch in duplex DNA by synthetic ligand-enhanced protein nanopore analysis

**DOI:** 10.1093/nar/gkae884

**Published:** 2024-10-16

**Authors:** Wenping Lyu, Jianji Zhu, XiaoQin Huang, Mauro Chinappi, Denis Garoli, Cenglin Gui, Tao Yang, Jiahai Wang

**Affiliations:** Department of Chemistry and Chemical Engineering, Guangzhou Key Laboratory for Environmentally Functional Materials and Technology, Guangzhou University, 230 Wai Huan Xi Road, Guangzhou Higher Education Mega Center, Guangzhou 510006, P.R. China; Department of Physics, RWTH Aachen University, Templergraben 55, 52062 Aachen, Germany; Department of Chemistry and Chemical Engineering, Guangzhou Key Laboratory for Environmentally Functional Materials and Technology, Guangzhou University, 230 Wai Huan Xi Road, Guangzhou Higher Education Mega Center, Guangzhou 510006, P.R. China; Department of Chemistry and Chemical Engineering, Guangzhou Key Laboratory for Environmentally Functional Materials and Technology, Guangzhou University, 230 Wai Huan Xi Road, Guangzhou Higher Education Mega Center, Guangzhou 510006, P.R. China; Univ Roma Tor Vergata, Dept Ind Engn, Via Politecn 1, I-00133 Rome, Italy; Istituto Italiano di Tecnologia, Via Morego 30, 16136 Genova, Italy; Dipartimento di Scienze e Metodi dell’Ingegneria, Università di Modena e Reggio Emilia, via Amendola 2, 42122 Reggio Emilia, Italy; Department of Chemistry and Chemical Engineering, Guangzhou Key Laboratory for Environmentally Functional Materials and Technology, Guangzhou University, 230 Wai Huan Xi Road, Guangzhou Higher Education Mega Center, Guangzhou 510006, P.R. China; Department of Chemistry and Chemical Engineering, Guangzhou Key Laboratory for Environmentally Functional Materials and Technology, Guangzhou University, 230 Wai Huan Xi Road, Guangzhou Higher Education Mega Center, Guangzhou 510006, P.R. China; Department of Chemistry and Chemical Engineering, Guangzhou Key Laboratory for Environmentally Functional Materials and Technology, Guangzhou University, 230 Wai Huan Xi Road, Guangzhou Higher Education Mega Center, Guangzhou 510006, P.R. China

## Abstract

Mismatched base pairs in DNA are the basis of single-nucleotide polymorphism, one of the major issues in genetic diseases. However, the changes of physical and chemical properties of DNA caused by single-site mismatches are often influenced by the sequence and the structural flexibility of the whole duplex, resulting in a challenge of direct detection of the types and location of mismatches sensitively. In this work, we proposed a synthetic ligand-enhanced protein nanopore analysis of GG mismatch on DNA fragment, inspired by *in silico* investigation of the specific binding of naphthyridine dimer (ND) on GG mismatch. We demonstrated that both the importing and unzipping processes of the ligand-bound DNA duplex can be efficiently slowed down in α-hemolysin nanopore. This ligand-binding induced slow-down effect of DNA in nanopore is also sensitive to the relative location of the mismatch on DNA duplex. Especially, the GG mismatch close to the end of a DNA fragment, which is hard to be detected by either routine nanopore analysis or tedious nanopore sequencing, can be well differentiated by our ND-enhanced nanopore experiment. These findings provide a promising strategy to localize and discriminate base mismatches in duplex form directly at the single-molecule level.

## Graphical Abstract

**Graphical Abstract:**
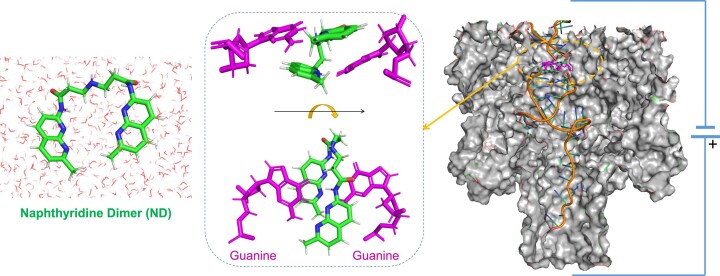

## Introduction

Mismatched base pairs in DNA are the foundation of single-nucleotide polymorphism (SNP), which are significant factors in genetic stability, disease progression and the development of therapeutic strategies (1–5). Detecting these mutations quickly can lead to timely interventions and treatments. There are still important efforts continuously made in the development of new and reliable analytical tools to investigate and detect mismatched base pairs. New techniques facilitating the above goal can have a profound impact on the field of personalized medicine. There are many efficient biosensors for DNA detection, including optical and electrochemical nanobiosensors (6), single DNA microarray (7), sequence terminus-dependent polymerase chain reaction (8), Clustered Regularly Interspaced Short Palindromic Repeats(CRISPR)-based method (9) and nanopore technology (10). Nanopore technology in particular demonstrates significant advantages in terms of accuracy and specificity, due to its single-molecule resolution. Protein nanopores (11), such as α-hemolysin (α-HL) (12), read double-stranded DNA (dsDNA) directly (10,13–17), providing a simplified detection workflow.

Transport of a dsDNA fragment though α-HL nanopore undergoes two major processes (13–15): (i) importing of the fragment in duplex form from solution to the central constriction in the β-barrel and (ii) unzipping of the duplex and transport of single-stranded DNA (ssDNA) through the central constriction in the β-barrel. The detection of mismatched base pair in a dsDNA heteropolymer using α-HL was demonstrated by analyzing the dwell time of the duplex within the pore (16,18), as the unzipping time is highly dependent on the thermal stability of the duplex. However, such analyses did not easily permit the discrimination of mismatched bases with similar thermodynamic and kinetic stabilities (e.g. CC versus CA), nor does the residence time typically reveal information about the position of the mismatch within the DNA sequence. In fact, the dwell time of a mismatched duplex in α-HL nanopore is generally shorter than that of a dsDNA without mismatches (16). The decrease in dwell time of mutated duplexes in the α-HL nanopore suggests that the total amount of information of the recorded blocking current signal is less than that of a well-paired DNA, which limits the feasibility of mutation detection from nanopore analysis.

On the other hand, synthetic ligands, such as naphthyridine dimer (ND), have been designed to recognize specific mismatches on dsDNA at nM scale (19–21). Surface immobilized ND can detect GG mismatch dsDNA from bulk solution sensitively (22), but it cannot differentiate between mismatch dsDNA fragments that contain different numbers and locations of mutations. Nevertheless, the binding of ND on GG mismatch dsDNA resulted in an increased melting temperature of the DNA duplex in solution (19–21), which suggests that the unzipping process of a GG mismatch dsDNA fragment in nanopore may also be prolonged by the ligand. The binding of ND on GG mismatch points may also cause steric hindrance to the importing of dsDNA in the pore. Such ligand-enhanced nanopore analysis of mismatch is a promising strategy, in principle, to extract more information from more recorded data of a prolonged dwell time.

Despite the DNA melting experiment, there is still a need for knowledge on the atomic details of the structural and dynamic properties of mismatched DNA fragments induced by ND. This knowledge is crucial in developing a robust GG mismatch detection technology via nanopore analysis. Limited by the sampling frequency of current experimental equipment, detailed transport process of a dsDNA fragment in nanopore is unavailable from the recorded ionic current signal. Herein, we combined molecular dynamics (MD) simulation and nanopore experiment. We investigated the binding behavior of ND on GG mismatch at different base pair step (bps) of a 15-bp DNA fragment by tens of microsecond-scale all-atom MD simulations. The latter enabled us to analyze the change of conformational fluctuations and hydrogen-bond (H-bond) kinetics of dsDNA induced by the binding of ND, and the subsequent transport behavior of two stable ND-bound dsDNA fragments (GG@bps7 and GG@bps12) in α-HL nanopore.

Our study shows that the binding of ND on GG mismatch DNA fragments is overall stable at microsecond timescale except when the mismatch is located at the two ends of DNA. For those stable ND-bound DNA (from GG@bps4 to GG@bps13), the overall structural rigidity and interstrand H-bond (interstrand-HB) stability of the DNA duplex are improved as a result. We then compared the transport behaviors of GG@bps7 and GG@bps12 in α-HL nanopore with and without the binding of ligand. MD simulations show that the latch region of the nanopore strongly confines both the importing and unzipping of ND-added DNA duplexes. In line with our simulations, the transport of ND-added mismatch DNA duplexes in nanopore experiments is significantly slower (more than one order of magnitude) than the case without the ligand. Both our simulation and experimental results demonstrate that the slow-down effect is sensitive to the location of GG mismatches, providing a promising strategy to localize the approximate position of GG mismatch by ligand-enhanced nanopore analysis.

## Materials and methods

### Setup of the simulation systems

Initial structure of the α-HL protein was obtained from the experimental structure deposited in Protein Data Bank (entry 7ahl) (12). Protonation state of the protein was assigned by the web server H++ assuming pH 7.0 (23). The protein was parameterized using amber14sb force field (24). The protein was embedded in a 12.5 nm × 12.5 nm dipalmitoylphosphatidylcholine (DPPC) bilayer membrane. The position of the protein within the bilayer was determined using the Orientations of Proteins in Membranes database (25). A 15-bp well-paired sequence (M0) of DNA model was generated online (https://novopro.cn/tools/random_dna.html). Then, 15 GG mismatched duplex (M1–M15) was assembled from two single-strand B-DNA using in-house code (Supplementary Code S1). All DNA models were parameterized using Parmbsc1 force field (26). The structure of simulated duplexes was constructed by B-DNA fragments generated by the fiber tool in X3DNA (27). The initial structure of ND molecule was optimized by Gaussian16 (28) program in gas phase, and then parameterized using the general AMBER force field (29) by antechamber tool (30) (Supplementary itp and pdb files). All the assembled nanopore complexes were solvated in a TIP3P (31) water box, with ion concentration of 1 M KCl. For each simulation, the system was energy minimized in 10 000 steps. Following the same protocol of our previous simulations of membrane protein (32), a series of restrained MD simulations were performed step-by-step to relax and equilibrate the protein and membrane system at 303.1 K and 1.0 bar using GROMACS (33) (version 2020.2).

### DNA analysis

We analyzed the interstrand HB of dsDNA in free solution (1 M KCl) by using the tool program hbonds of GROMACS package. H-bonds were determined based on cutoffs for the hydrogen–donor–acceptor angle less than 30° and the donor–acceptor distance shorter than 0.35 nm. The distribution of the number of interstrand HB is fitted to a Gaussian distribution (Supplementary Table S1) by using curve_fit module of scipy (34) package. Although the duplex structure is well maintained in all simulations, the interstrand HB of DNA is not always formed due to structural fluctuations. Thus, we characterized the stability of the base pairing by the continuous (uninterrupted) lifetime (35,36) of the interstrand HB, which measures the mean first passage time of all newly formed H-bonds between the two strands of DNA. The error of interstrand-HB lifetime of each system is estimated by block averaging. Here, each block of the MD trajectory is 100 ns long and contains 10 000 frames.

### Free energy calculation

Umbrella sampling and weighted histogram analysis method were employed to estimate the potential of mean force (PMF) of the translocation free energy profiles. A harmonic potential (37) was applied on the *z*-direction component of the center of mass (COM) of the duplex, which is defined as the reaction coordinate of DNA importing. To avoid the drift of the nanopore system during the sampling, backbone atoms of the protein and membrane were fixed. More than 90 sampling windows were employed in each PMF calculation, covering the reaction coordinate from 3.5 to 9 nm. Fifty nanoseconds of umbrella sampling was performed for each sampling window, to ensure the overlap between umbrella histograms (Supplementary Figure S1). The PMF was reconstructed by using g_wham program (38), and the error was estimated using the Bootstrapping protocol (38). Wilcoxon rank sum test was performed to estimate the statistical significance of the observed difference of free energy.

### Simulation of DNA duplex unzipping in nanopore

The unzipping of DNA duplex in nanopore is a nonequilibrium process requiring external force. To estimate the required external force of DNA unzipping, we performed steered MD simulation (39) with a constant pulling velocity (0.001 nm/ps) of the terminal base of one strand of the DNA duplex. To avoid the drift of the nanopore system, backbone atoms of the protein and membrane were fixed. Then, an averaged force spectrum was obtained from the averaged pulling force of 25 individual nonequilibrium simulations. The statistical error is defined as the standard error estimation of the 25 recorded pull forces. We calculated the averaged force spectrum of four DNA duplexes unzipping in the nanopore: GG@bps7, GG@bps7 + ND, GG@bps12 and GG@bps12 + ND, with the starting conformations from the end states (reaction coordinate >9 nm) of each PMF calculation. Wilcoxon rank sum test was performed to estimate the statistical significance of the observed difference of rupture force (Supplementary Table S2).

### Simulation of DNA duplex unzipping in free solution

As a comparison, we calculated the force spectrum of duplex unzipping of GG@bps7, GG@bps7 + ND, GG@bps12 and GG@bps12 + ND in free solution, respectively. The starting conformation of the four duplexes were taken from the end states (reaction coordinate >9 nm) of each PMF calculation, and then separately solvated in a TIP3P (31) water box (11.4 × 9.9 × 30 nm^3^), with ion concentration of 1 M KCl. Fifty nanoseconds of restraint MD was performed to relax the solvent of each system, after an energy minimization of 5000 steps. Same as the DNA unzipping simulations in nanopore, we performed steered MD simulation (39) with constant pulling velocity (0.001 nm/ps) of the terminal base of one strand of the DNA duplex. Note that during the separation of DNA in nanopore, the complementary chain of DNA will be obstructed by the bottom of the nanopore. To mimic the blockage of the complementary chain by the nanopore, we introduced an anchor point of the complementary chain of DNA during the pull simulation in free solution. For instance, the position of the terminal base of the other strand of the duplex is fixed during simulation (anchor point; Supplementary Figure S2A). This setup allows for a clear observation of the unzipping behaviors of DNA without the confinement of nanopore chamber. Similarly, each force spectrum was averaged from 25 individual nonequilibrium simulations. The statistical error is defined as the standard error of the 25 recorded pull forces.

### Materials of nanopore experiment

Lipid 1,2-diphytanoyl-*sn*-glycero-3-phosphocholine (DPhPC) was purchased from Avanti Polar Lipids (Alabaster, AL, USA). Wild-type α-HL for the formation of nanopore was gifted from Dr Wu’s lab at Chinese Academy of Sciences. DNA oligonucleotides were synthesized and purified with high-performance liquid chromatography by Sangon Biotech Co. Ltd (Shanghai, China). All the other chemical materials were obtained from Aladdin Biochemical Technology Co. Ltd (Shanghai, China).

### DNA preparation

All DNAs were dialyzed against 10 mM potassium phosphate buffer (pH 6.2) for >12 h. The duplex probe was prepared in hybridization buffer (10 mM Tris, 100 mM NaCl, pH 7.4). The solution was incubated at 90°C for 5 min and then was allowed to cool to room temperature.

### Nanopore electrical recording

Planar lipid bilayer membranes by DPhPC were formed by applying DPPC (10 mg/ml) in decane to a 100-μm orifice in a 0.5-ml bilayer chamber filled with an electrolyte solution. The α-HL protein (5 μg/ml) was added into the *cis* chamber, which was connected to ‘ground’. The potential was applied from the *trans* chamber by two Ag/AgCl electrodes at 200 mV. Once a single α-HL nanopore was inserted in the lipid bilayer, the probes, target DNA or their mixtures were added to the *cis* chamber at a final concentration of 200 nM. Currents were recorded with a patch clamp amplifier (Axopatch 200B, Axon Instruments, Foster City, CA, USA). The signal was low-pass filtered at 2.9 kHz and sampled at a frequency of 50 kHz with a Digidata 1550B A/D converter (Axon Instruments, Foster City, CA, USA). All data were recorded in 1.0 M KCl (10 mM Tris, 0.1 mM EDTA, pH 8.0) solution at +120 mV and 22 ± 2°C.

### Experimental data analysis

Single-channel data were acquired and analyzed using the Axon™ pClamp™ 10.6 software (Molecular Devices). We described the normalized current blockages as d*I*/*I*_0_, where *I*_0_ is the open pore current and d*I* = *I*_0_ − *I* is the current blockage generated by the translocation of the analysts. Events with d*I*/*I*_0_ larger than 88% were counted for dsDNA, which were attributed to the polymer translocation through the nanopore. The median dwell time of signal events was obtained from the data points of dwell time within the 95% confidence interval of the best-fit Gaussian function applied to the histograms of all data points.

## Results and discussion

### ND binding on GG mismatch at different locations of DNA duplexes

**Figure 1.**
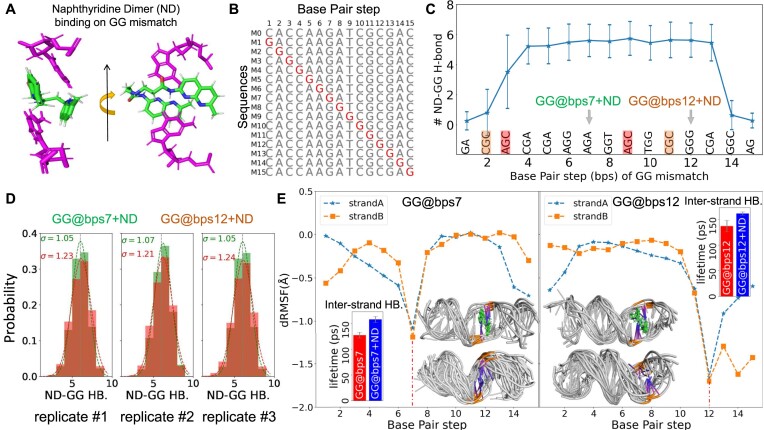
(**A**) Structure of ND (color code: green) binding on GG mismatch (color code: magenta). (**B**) Sequence of the well-paired DNA and the 15 GG mismatch models. The GG mismatch point of each model is highlighted in red. (**C**) The number of ND–GG H-bonds of the 15 GG mismatch models. The error bar of each data point represents the standard deviation of the calculated number of ND–GG H-bonds of 300 000 structures obtained from three independent MD simulations (100 000 structures each, with time interval of 10 ps). Local sequence environment surrounding the GG mismatch is annotated in the bottom of plot. The local sequence environments of GG@bps7 and GG@bps12 are highlighted by arrows. (**D**) The distribution of the number of ND–GG H-bonds observed in three independent simulations of GG@bps7 + ND (green) and GG@bps12 + ND (red), respectively. The dot line curves are the best Gaussian fit of each distribution. (**E**) Comparison of the ND binding induced dRMSF of the GG@bps7 and GG@bps12, respectively. Insets report the continuous lifetime (36) of H-bonds (d*t*= 10 ps) between two stands of the duplexes and the representative conformations of the two duplexes with and without the addition of ND, respectively.

ND is known to recognize GG mismatch in duplex DNA strongly and selectively, due to intermolecular H-bond and stacking interactions (Figure 1A). However, the relation of the binding stability and the relative location of mismatch on DNA fragment is not systematically studied yet. Here, we studied the binding behavior of ND on 15 model DNA fragments (M1–M15; Figure 1B); each of them contains a single-point GG mismatch at different bps. The DNA models were 15 bp long, so the mismatch covered all bps of the duplex. These models included nine different local sequence environments (3-mer) surrounding the GG mismatch on DNA: CGC, AGC, CGA, AGG, AGA, GGT, TGG, GGG and GAC. The initial binding structure of ND on the model DNA fragments was obtained by aligning the ND + GG motif with a previous experimental structure (40). We then performed all-atom MD simulations for each DNA–ND complex in solution with three independent replicas. For comparison, we also carried out the same amount of MD simulations for each of the 15 mismatch models without the addition of ND and for a well-paired DNA model (M0; Figure 1B) as the common reference.

The binding behavior of ND on GG mismatch is characterized by counting the number of H-bonds between ND and GG mismatch (ND–GG H-bond) in simulations. We find that the number of ND–GG H-bonds is quite stable (in microsecond timescale) for most of these mutation positions (Figure 1C), except for the mismatch at the two ends of DNA fragment (GG@bps1, GG@bps2, GG@bps3, GG@bps14 and GG@bps15). Interestingly, both GG@bps2 and GG@bps11 share the same local sequence environment of ‘CGC’ (highlighted in yellow; Figure 1C); however, stable binding of ND is observed only at GG@bps11. A similar difference is noted between GG@bps3 and GG@bps9 (highlighted in red; Figure 1C). Thus, the ND cannot bind stably to the GG mismatch at the first or last one to three base pairs of the DNA fragment, regardless of the local sequence environment surrounding the mutation.

Next, we selected GG@bps7 and GG@bps12 from the GG mismatch fragments that exhibit stable ND binding, using them as two models to represent mismatches located in the middle and at the end of the DNA fragment, respectively. We investigated the details of ND–GG binding in the six independent simulations of the GG@bps7 and GG@bps12 (Figure 1D). The distribution of ND–GG H-bonds is centered at 6 (noted as dashed line), indicating that the ND–GG binding pattern is well conserved during these simulations at microsecond timescale. However, the height of the distribution peak of GG@bps7 + ND (green) is higher than that of GG@bps12 + ND (brown). Indeed, the best (height fixed) Gaussian fit of the distributions shows that the width of the fitted Gaussian distribution of GG@bps7 + ND is always smaller than the value of GG@bps12 + ND (Supplementary Table S1). Smaller the width of the distribution, narrower is the fluctuation range of the number of ND–GG H-bonds. In other words, the binding of ND on GG mismatch located at the middle of a DNA fragment is more stable than when it is positioned near the end. Notably, the local sequence environment of GG@bps7 is ‘AGA’, which is anticipated to be less binding affinity of the recognition of ND by the ‘GGG’ motif in GG@bps12. We thus propose that the unusual difference in ND–GG H-bond distribution between the two models is primarily due to the location of the GG mismatch, which results from significant structural fluctuations inherent at the end of DNA for both well-paired and mismatched duplexes (Supplementary Figure S3).

The binding of ND improves the overall structure rigidity of both DNA fragments, as indicated by the negative difference in root-mean-square fluctuation (dRMSF) of each DNA base with and without the binding of ND (Figure 1E). The most significant drops in dRMSF are observed at the mismatched base pair (7th bps and 12th bps), where ND is bound. The higher the structure rigidity, the lower is the range of structure fluctuation. Indeed, only five and six representative conformations for the GG@bps7 + ND and GG@bps12 + ND are obtained from the simulation trajectories (RMSD cutoff = 2 Å) (33), respectively (insets of Figure 1E). In contrast, tens of representative conformations are obtained for the two DNA models without the stabilization of ND at the same clustering criteria (33) (insets of Figure 1E). Although the overall interstrand base pairing of all four systems is well maintained at microsecond timescale, the averaged uninterrupted lifetime (35) of H-bonds between the two DNA strands is prolonged in the binding of ND (Figure 1E). It implies a slowed-down kinetics of duplex unzipping or an elevated energy barrier of DNA melting.

In nanopore analysis, the dwell time and the blocking current of each transport event are highly sensitive to the conformation change, and thermal and chemical stability of translocating molecules (41–46). All the aforementioned findings indicate that the binding of ND resulted in a mismatch location-dependent change in both the structural and dynamic behavior of the DNA fragments. In particular, the addition of ND induced improved structural rigidity and enhanced interstrand-HB interaction, which might prolong the overall dwell time of the duplex inside an α-HL nanopore, facilitating the study of the GG mismatch on DNA duplex.

### The effect of ND on the importing of DNA into α-HL nanopore

**Figure 2.**
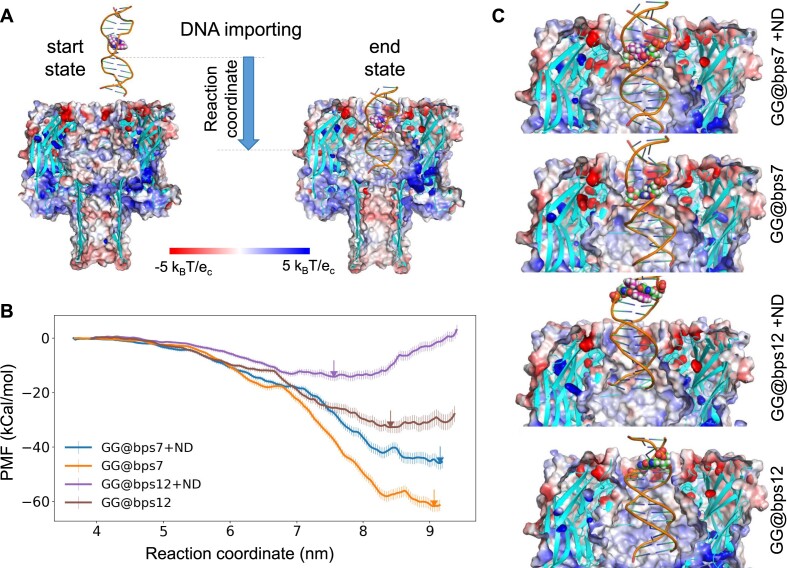
(**A**) The DNA importing process considered in the free energy calculation. Here, the start and end state of this process is shown for GG@bps7 + ND. The protein is shown as colored surface model according to the electrostatic potential surface. The GG mismatch and ND are highlighted in the sphere model. The reaction coordinate is defined as the *z*-component of the COM coordinate of the DNA duplex. (**B**) The impact of ND on the PMF of import DNA duplex to α-HL nanopore. The minimum free energy point (MEP) of each PMF is marked by arrows. (**C**) The representative conformations of DNA at the MEP. The ND and GG mismatches are highlighted by the sphere model.

To elucidate the impact of ND binding on the in-pore process of DNA, we conducted PMF calculations of the importing of the DNA in duplex form into α-HL nanopore (Figure 2) using umbrella sampling (37,38,47,48) (see the ‘Materials and methods’ section). The *z*-component of the COM of the DNA fragment was chosen as the reaction coordinate in calculation (Figure 2A). By this definition, no external force is directly applied on the ND molecule during the umbrella sampling. Here, we aimed to capture the free energy contributions of purely intermolecular interactions; thus, the PMF calculations were performed without the interference of external electric field (37). In accordance with the previous models, we compared the free energy profiles of four systems: GG@bps7, GG@bps7 + ND, GG@bps12 and GG@bps12 + ND (Figure 2B). Results indicate that the DNA duplexes undergo a free energy trap during the in-pore translocation (Figure 2B), which suggests the presence of electrostatic attractions between the negatively charged DNA and the positively charged cavity inside the protein (Figure 2A).

The depth of the free energy traps at the lowest MEP is reduced by the binding of ND, and this reduction depends on the location of the GG mismatch (Figure 2B). The deepest free energy trap is observed for the two systems with GG mismatch at the middle of duplex (GG@bps7 and GG@bps7 + ND, about −60 and −45 kcal/mol, respectively) when the duplexes are fully captured into the nanopore (Figure 2C). In contrast, the GG@bps12 + ND system exhibits the highest MEP when the ND-bound GG mismatch is still located outside of the pore (Figure 2C). For the GG@bps12 + ND system, a free energy barrier raised rapidly during the shift of the ND-bound GG mismatch toward the latch region of the nanopore (Figure 2B). It indicates a clear hindrance effect of the specific interaction between the latch region of the nanopore and the mismatch of the duplex on the in-pore translocation process.

Considering the identical net charge of the DNA duplex and the protein across all four systems, the deeper free energy traps at the MEP are indicative of more ‘relaxed’ DNA duplex structures within the nanopore’s confined space that establish more stable electrostatic attraction between the DNA backbone and the positively charged surface at the bottom of the nanopore chamber. Thus, the differentiated importing behaviors of the four systems are the result of varying deformation energy of DNA (intramolecular) and protein–DNA (intermolecular) interactions. Both types of interactions are sensitive to the mismatch location on the DNA duplex and can be significantly adjusted by the binding of the ligand ND.

### The impact of ND on the unzipping of DNA duplex in α-HL nanopore

**Figure 3.**
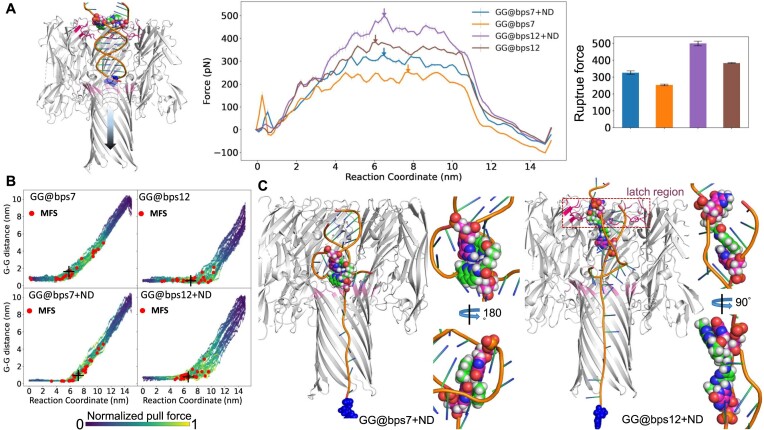
(**A**) A demo of applied pull force on the terminal base of strand A of a DNA fragment (left panel); the calculated force spectrum of DNA unzipping in α-HL nanopore and the estimated rupture force of each system. The error bars represent the standard error estimation of the force spectrum, derived from 25 independent replica simulations. (**B**) The correlation between the separation of GG mismatch (G–G distance) and the *z*-component of COM of the base of pull of each independent nonequilibrium simulation. The data points are colored with the normalized pull force. The red disks are the MFS of each simulation. The MFS most close to the averaged value of all replicas is marked by black cross for each system. (**C**) Representative conformation of the GG@bps7 + ND and GG@bps12 + ND at the MFS (panel B). Detailed stacking structures of ND on the two mismatched guanines are highlighted in the right panel for each system. The protein nanopore is shown in cartoon model. The terminal base under pull is highlighted in the sphere model.

Another important in-pore process of dsDNA transport in α-HL nanopore is the unzipping of the two strands of the duplex (13,14,16,18), which requires breaking tens of H-bonds. The binding of ND improves the strength of these H-bonds in solvent, as mentioned in the previous section. In nanopore experiments, a strong pulling force, either from an electric field across nanopore or an external force (13) such as optical tweezers (49,50), is required to rupture the interstrand interaction within the duplex. Here, we characterized the impact of ND binding on such a nonequilibrium process by simulated force spectrum (50–54). To simulate the force spectrum, a pull force with constant pulling velocity of 0.001 nm/ps was applied on the terminal nucleotide of the 5′-end of DNA strand A (Figure 3A). A force spectrum was obtained as the average of pull force recorded in 25 replicas of such nonequilibrium simulations.

The force spectrum of the DNA duplex unzipping in the nanopore exhibits three stages (right panel; Figure 3A): a slow force increase process starting around the zero-force point, a stable high force plateau and a force release process. These stages correspond to the deformation of the duplex structure, interstrand interaction breaking and the release of the unzipped ssDNA chain to *trans* chamber, respectively (Supplementary Movie S1). In the following, we will indicate the maximum force state (MFS) on the force spectrum as the rupture force. We find that the binding of ND on the GG mismatch significantly improves the rupture force of the DNA duplex in the nanopore (*P*-value <0.05; Supplementary Table S2), regardless of whether the GG mismatch is in the middle or at the end of a DNA fragment. This observation is consistent with the stabilized interstrand interaction of ND-bound dsDNA as observed in solvent (Figure 1).

In addition to the enhanced rupture force on average, the range of the distribution of the MFS along the reaction coordinate of each nonequilibrium simulation is reduced by the binding of ND (Figure 3B). Furthermore, the MFS is more frequently observed around the separation point of the two mismatched guanines for both GG@bps12 + ND and GG@bps7 + ND (Figure 3B). The representative conformation of ND at these MFS is stretched intensively by the stacking attraction from the two mismatched guanine (Figure 3C and Supplementary Figure S4). Therefore, the separation of the mismatched base pair with ND, which is supposed to be the most energetic favorable without ND, might become the determining step of DNA unzipping in the nanopore.

The highest rupture force (∼500 pN) is observed for GG@bps12 + ND, which is around 150 pN larger than the value of GG@bps7 + ND (Figure 3A). At the MFS of GG@bps12 + ND (Figure 3C), the ND + GG motif is just across the latch region (colored in hot pink inside a red dashed box), which has the smallest cavity on the upper half of the α-HL nanopore. Comparing the GG mismatch at different locations of DNA fragment, the stronger the confinement of nanopore on the GG mismatch point (closer to the latch region or smaller cavity), the narrower the distribution range of the MFS on the reaction coordinate of the unzipping pathway (Figure 3B). Thus, we propose that the confinement effect of the nanopore on the DNA duplex significantly hinders the unzipping process.

To demonstrate the existence of the above effects of nanopore confinement, we calculated the force spectrum of duplex unzipping of both ND-hybrid and ND-free DNA fragments in free solution (Supplementary Figure S2). We find that the rupture force of the unzipping of the duplexes in free solution is only ∼80 pN, which is slightly larger than the value of the reported rupture force (∼60 pN) of a well-paired DNA fragment in the previous single-molecule experiment (55). Notably, during the pulling, the DNA models in free solution underwent a rotation before the unzipping of the duplex (Supplementary Figure S2). These results indicate that the observed strong rupture force (300–500 pN) of either ND-hybrid or ND-free DNA unzipping in nanopore is mainly due to the confinement of nanopore: the rotation of DNA is not allowed in nanopore. As the nanopore limits the spatial freedom of the DNA duplex, this confinement affects the dynamics of DNA unzipping.

Taken together, the binding of ND on GG mismatch will slow down the DNA unzipping and reduce the randomness of the dynamics of the separation of mismatch point in nanopore, which relies on both the direct ND–GG binding and the mismatch location-dependent nanopore confinement.

### Wet-lab performance of ND-enhanced nanopore analysis on GG mismatch

**Figure 4.**
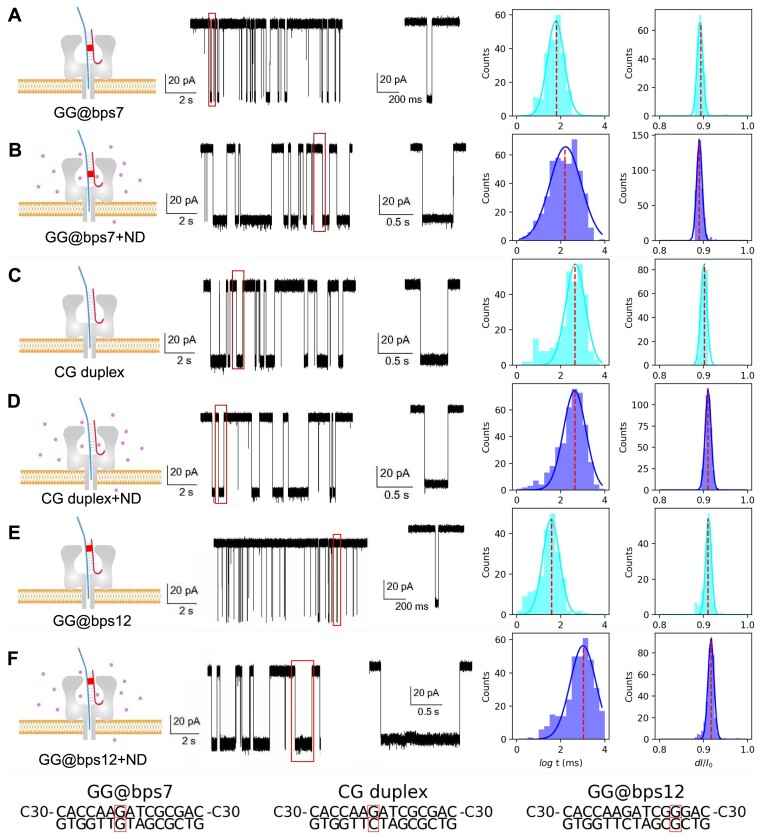
The recorded ionic current of the GG@bps7 (**A**, **B**), well-paired CG duplex (**C**, **D**) and GG@bps12 (**E**, **F**) in a nanopore experiment. The ND is shown as dots in panels (B), (D) and (F). The distribution of dwell time [log *t* (ms)] and the relative dwell current (d*I*/*I*_0_) are showed in the right plot of panels (A)–(F), respectively. The distribution peak of dwell time and d*I*/*I*_0_ are marked by dashed lines. The sequences of each chain of different DNAs used in experiments are reported in the bottom.

Finally, we conducted nanopore experiment to evaluate the wet-lab performance of ND-enhanced nanopore analysis of GG mismatch (Figure 4). To improve the capture rate of DNA from solvent to nanopore, the pulled DNA strand in aforementioned force spectrum calculation is linked with a (C30) guide sequence: 5′-C30-CACCAAGATCGCGAC-C30-3′ for GG@bps7 and 5′-C30-CACCAAGATCGGGAC-C30-3′ for GG@bps12, respectively.

For the model of GG mismatch in the middle of DNA fragment (GG@bps7), the current time trace shows normal event signatures with a median value of dwell time of 65.45 ± 4.41 ms (Figure 4A). By using the GG@bps7 + ND complex, a much longer (*P*-value <0.05; Supplementary Table S3) duration with a median dwell time of 163.41 ± 22.42 ms is observed (Figure 4B). We also compared the impact of ND on the corresponding well-paired DNA duplex of GG@bps7, CG duplex, by nanopore experiment (Figure 4C and D). The median values of event duration for samples before and after the addition of ND almost did not change significantly (447.7 **±** 37.71 and 451.37 ± 34.43 ms, *P*-value >0.05; Supplementary Table S3). These results consolidate that the specific binding between the ligand ND and the GG mismatch will slow down the in-pore processes of DNA duplex.

Next, we verified the model of GG mismatch in the end of DNA fragment (GG@bps12): the median of dwell time is only 40.0 ± 2.68 ms (Figure 4E). This value is shorter (*P*-value <0.05; Supplementary Table S3) than the corresponding value of GG@bps7, on which the mismatch point is in the middle of the chain. The shorter the dwell time, the more challenging it is to perform a reliable nanopore analysis. As discussed in the analysis of the interstrand interaction of GG@bps12 in solvent (Figure 1), such a short dwell time might be caused by the inherent structure fluctuation at the end of a DNA fragment, which is also a bottleneck of many other nanopore sequencing/detection technologies. However, we also noticed that before unzipping, the GG mismatch of GG@bps12 is located closely to the latch region of α-HL nanopore (Figure 3A), which is the narrowest part during the transport of the mismatched DNA in duplex form in the nanopore and it has been reported to be sensitive to the discrimination of local conformations of dsDNA (56), especially those involving flipping base pairs (57) and abasic site (58). The relatively short dwell time observed for the GG@bps12 means that without the ligand, even the GG mismatch is located at the most sensitive region, the α-HL nanopore is still not able to block the transport of DNA efficiently.

In line with our simulated force spectrum where we observed that the GG@bps12 + ND requires the highest rupture force during DNA unzipping, the median dwell time of GG@bps12 + ND (Figure 4F) is 1068.0 ± 110.79 ms, over 25 times more with respect to the case without the ligand (GG@bps12; Figure 4E). The dwell time of GG@bps12 + ND is even longer than the case of GG@bps7 + ND (Figure 4B, *P*-value <0.05 and Supplementary Table S3), which means that the slow-down effect of ND on DNA transport in α-HL nanopore is most significant when the ND–GG motif is located close to the latch region of nanopore before the unzipping of the duplex.

In short, these results are a proof of concept of how the performance of α-HL nanopore analysis can be significantly modulated by playing with the specific binding of a ligand on mismatch point of DNA and the ligand-binding enhanced nanopore confinement. The underlying mechanism of mismatch locating is a position matching between the ligand-binding point and the latch region, which is most sensitive before the unzipping of the duplex.

On the other hand, by the addition of ND, there is also some overlap between the distributions of dwell time of well-paired CG duplex (Figure 4C and D) and ND-bound GG duplexes (Figure 4B and F). It means that the prolonged dwell time by the ND-enhanced nanopore analysis might need to be further improved in detection applications. Our understanding of the underlying mechanism of ligand-enhanced mismatch locating above could be beneficial in the next stage of researches on the protein engineering of the nanopore and modification of the ligand with precisely controlled dwell time.

## Conclusion

In summary, we proposed a nanopore analysis strategy of GG mismatch by introducing the synthetic ND ligand. We systemically investigated the structure and dynamics of ND binding on GG mismatched DNA fragments with different mutation locations. The stable ND–GG interaction in combination with the confinement effect of nanopore induced distinct hindrance on the translocation and slowed down the unzipping process.

Furthermore, by using the ligand ND, this confinement effect is significantly enlarged for GG mismatch close to the end of DNA fragment, as observed in both simulated force spectrum and recorded dwell time. This is a result of position matching of the ND bound GG mismatch and the latch region of α-HL nanopore. These findings indicate that the α-HL nanopore is promising to detect and locate GG mismatch by the aid of the synthetic ligand ND. This strategy can be easily extended to other ligands to detect different types of mismatches and pave the way for advanced methodology in SNPs detection based on biological nanopores.

## Supplementary Material

gkae884_Supplemental_Files

## Data Availability

The data underlying this article are available in the article and in its online supplementary material.

## Notes

Present address: Wenping Lyu, School of Medicine, Chinese University of Hong Kong, Shenzhen Campus, Shenzhen 518172, China.
