# Supplementary material for: Localization and discrimination of GG mismatch in duplex DNA by synthetic ligand-enhanced protein nanopore analysis
Source: Nucleic Acids Res. 2024 Oct 16;52(20):12191–200. doi: 10.1093/nar/gkae884 (PMC11551735; doi:10.1093/nar/gkae884)
Supplement: gkae884_Supplemental_Files [file gkae884_supplemental_files.zip › SI_SUB_0825.pdf]

Supplementary information:

Localization and discrimination of GG mismatch in duplex DNA by synthetic ligand-enhanced protein nanopore analysis

Wenping Lyu[a, b,+], Jianji Zhu[a], XiaoQin Huang[a], Mauro Chinappi[c], Denis Garoli[d,e], Cenglin Gui[a], Tao Yang[a], Jiahai Wang*[a]

[a] Department of Chemistry and Chemical Engineering, Guangzhou Key Laboratory for Environmentally Functional Materials and Technology, Guangzhou University, Guangzhou 510006, P.R. China

[b] Department of Physics, RWTH Aachen, Templergraben 55, 52062 Aachen, Germany

[c] Department of Industrial Engineering, University of Rome Tor Vergata, 00133 Rome, Italy

[d] Istituto Italiano di Tecnologia, Via Morego 30, 16136 Genova, Italy.

[e] Dip. di Scienze e Metodi dell'Ingegneria, Università di Modena e Reggio Emilia, via Amendola 2, 42122 Reggio Emilia, Italy

* Corresponding author: E-mail: jiahaiwang@gzhu.edu.cn,Tel: +86-18816801579

+ Current address: School of Medicine, Chinese University of Hong Kong, Shenzhen campus, Shenzhen 518172, China.

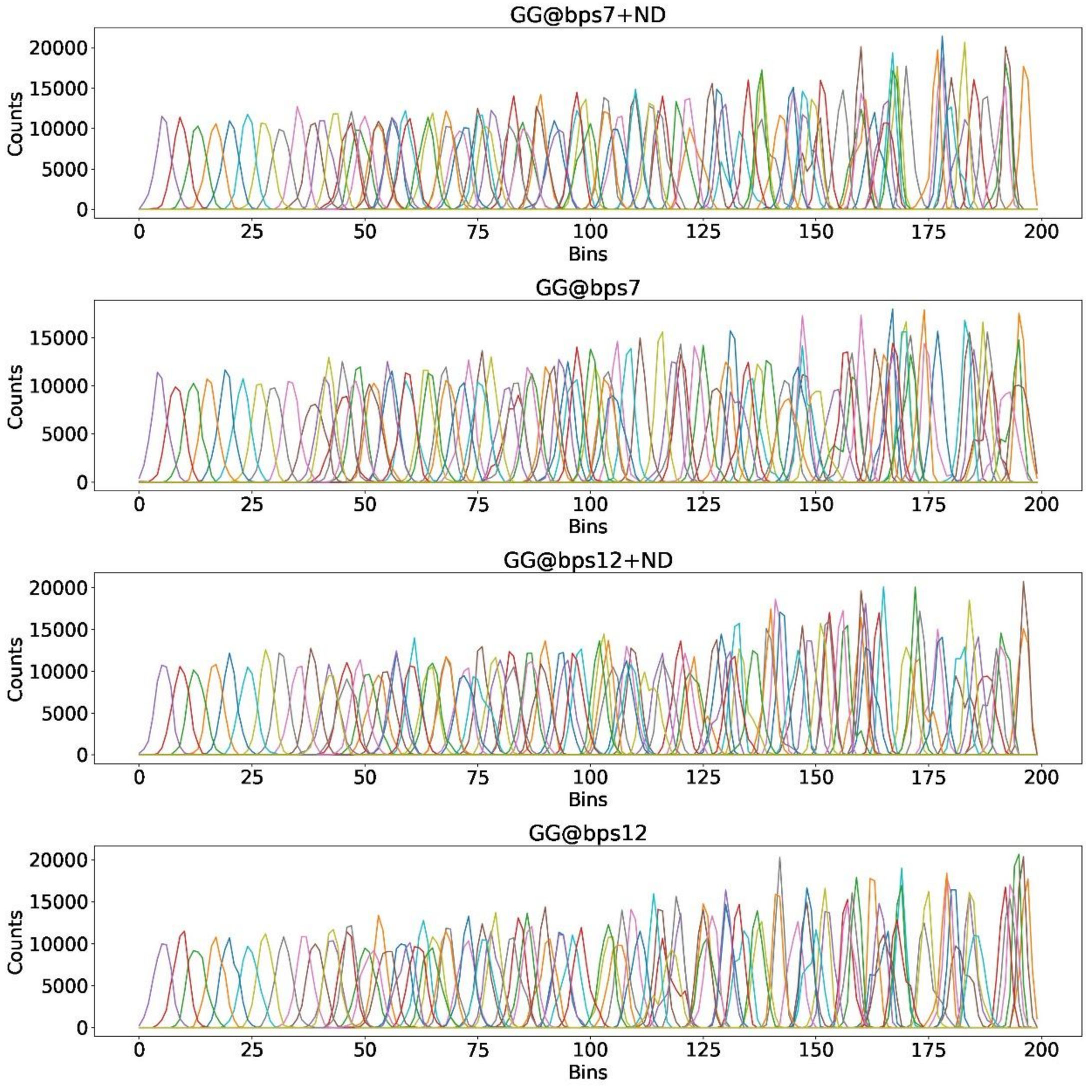

Fig. S1 Histogram distribution of the umbrella potentials recorded in each umbrella sampling window of GG@bps12, GG@bps12+ND, GG@bps7, and GG@bps7+ND. For each umbrella window, we performed 50 ns of umbrella sampling to ensure the overlap between umbrella histograms. More than 90 sampling windows in each PMF calculation.

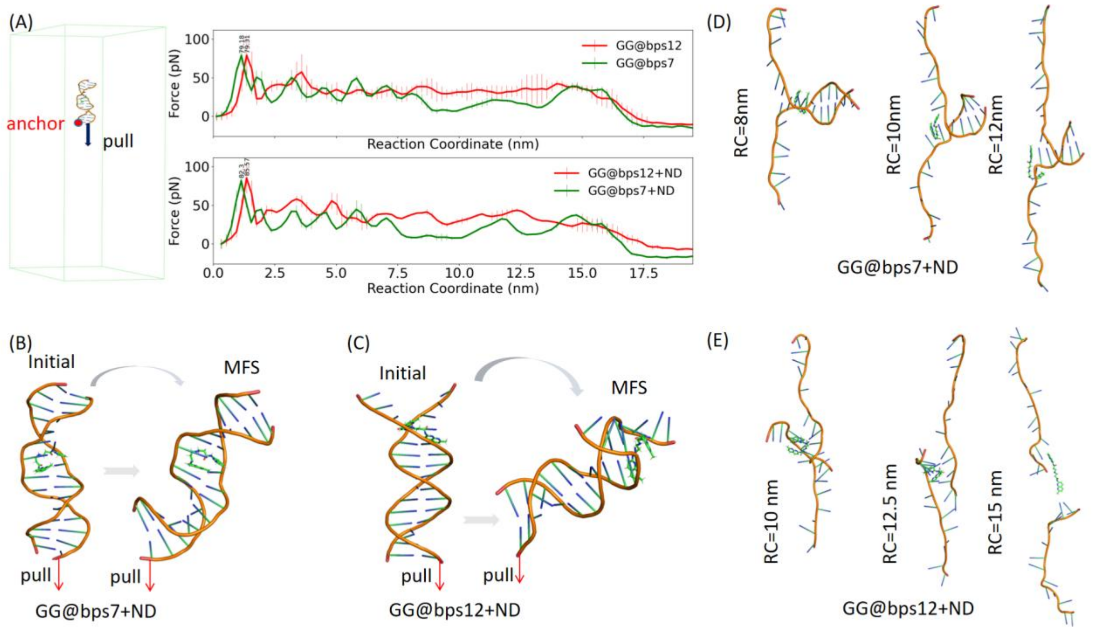

Fig. S2 (A) The calculated force spectrum of GG@bps12, GG@bps7, GG@bps12+ND, and GG@bps7+ND in free solution. The location of maximum force state (MFS) on the force spectrum are annotated. Schematic diagram of the set-up of anchor and pull points in MD simulation is shown as the left panel. (B-C) The conformation transition of the ND-hybrid DNA duplexes from the initial state to the MFS. It shows that the MFS is relevant to the rotation of the duplex upon pulling. Here, the DNA is rendered in cartoon model and the ligand ND is rendered in sticks model. (D) The snapshots of the GG@bps7+ND unzipping around the mismatch point (RC~9 nm) are shown. The reduced pull force between RC=8 nm to RC=10 nm suggests that the base-pairing around the GG mismatch is less stable than the well-paired part of the duplex (RC<7 nm). However, the breaking of the ND+GG motif requires a significant increase of the pull force (from RC=10 nm to RC=12 nm), indicating the ND-induced slowdown effect on the duplex unzipping. (E) The snapshots of the GG@bps12+ND unzipping around the mismatch point (RC~10 nm) are shown, revealing an upside-down inter-strand orientation. This suggests that the GG mismatch at the end of the DNA fragment is much more unstable in free solution than when the mismatch is in the middle of the DNA (GG@bps7+ND). Regardless, the ligand ND is always involved in the separation process of the two strands (RC from 10 nm to 15 nm).

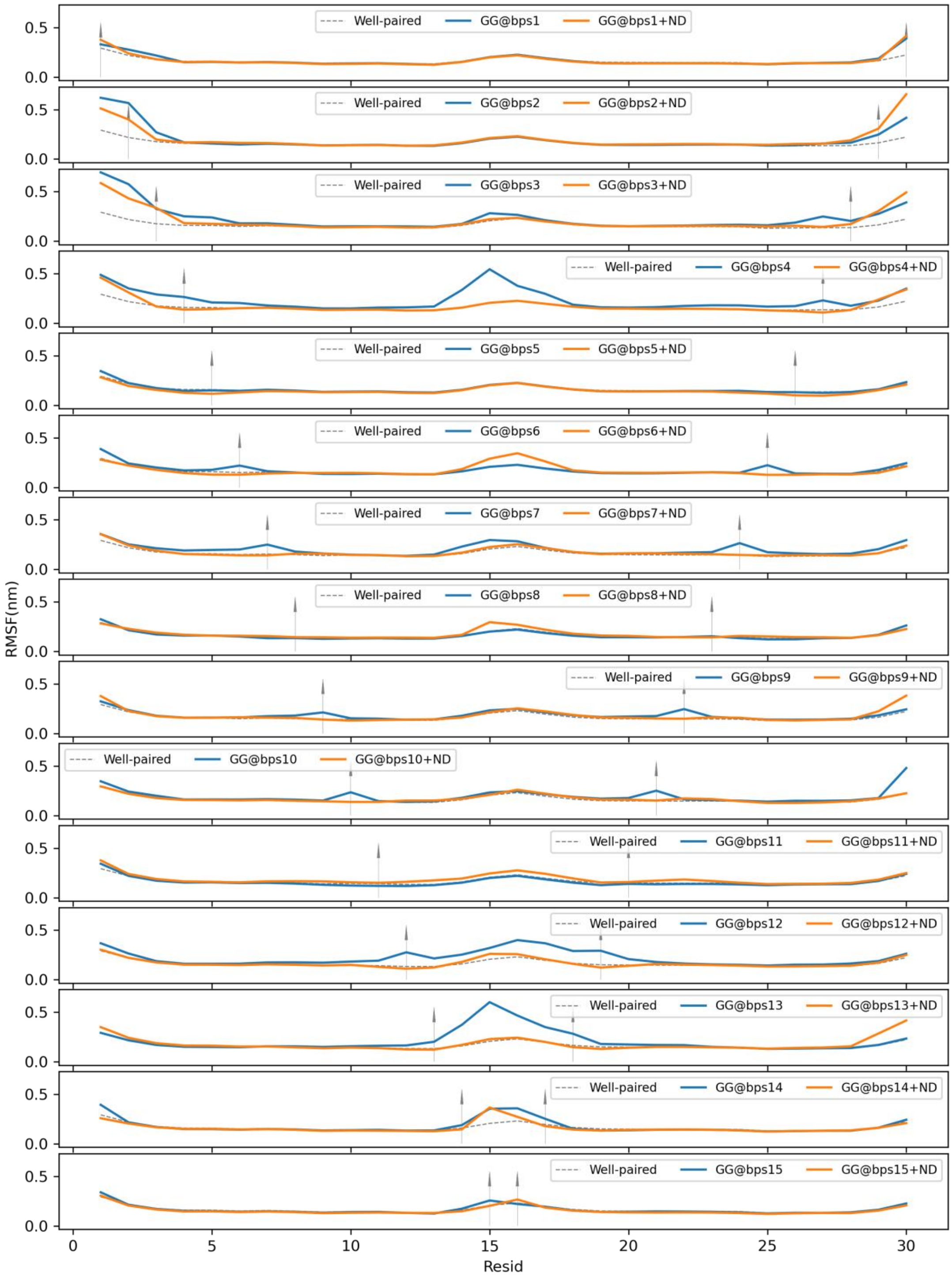

Fig. S3 Comparison of the impact of GG mismatch and the binding of ligand ND on the root-mean-square fluctuation (RMSF) of DNA models. The RMSF of the well-paired DNA is represented as dotted line. The position of GG mismatch is highlighted by arrows for each model.

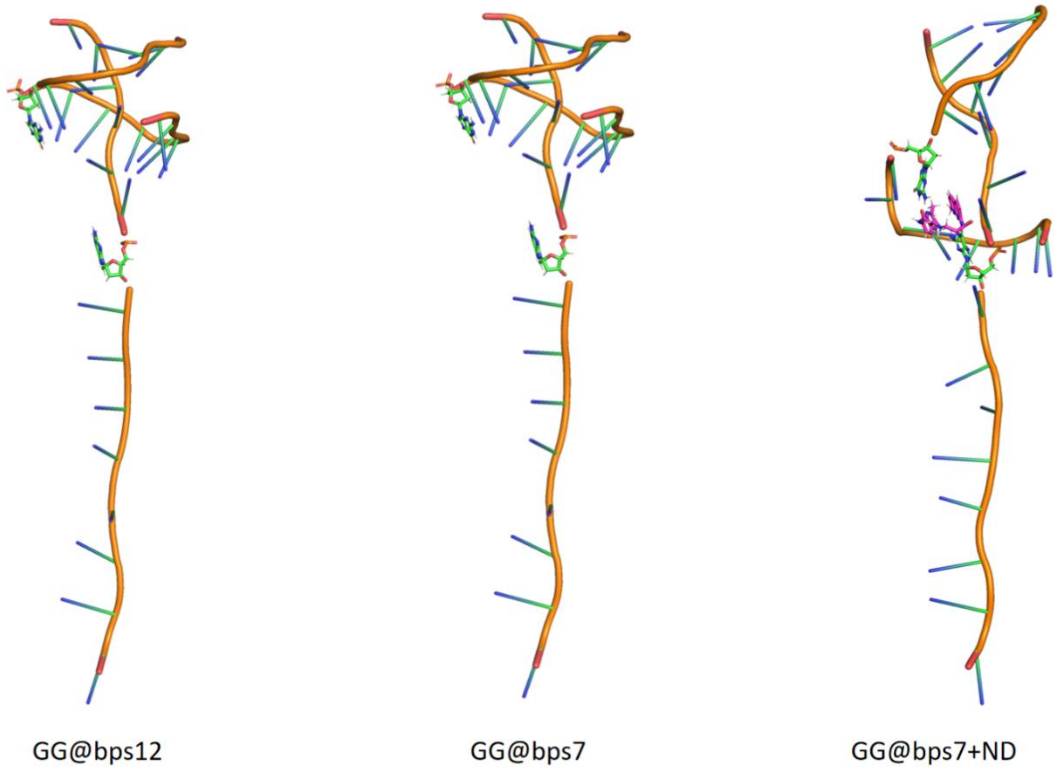

Fig. S4 Representative conformations of the GG@bps12, GG@bps7, and GG@bps7+ND at maximum force state (MFS). The DNA duplex is rendered in cartoon model, but the GG mismatch is rendered in sticks model. The ligand Naphthyridine Dimer (ND) is rendered in sticks model and highlighted in color *magenta*.

Table S1. Non-standard parameters used for the Gaussian model fitting in main text Fig. 1. Parameters Y-max, μ and σ represent the height, center and width of the best fitted Gaussian curve, respectively.

	Y-max	μ	σ
GG@bps7 #1	0.38	6.03	1.05
GG@bps7 #2	0.37	6.22	1.07
GG@bps7 #3	0.38	6.02	1.05
GG@bps12 #1	0.33	6.12	1.23
GG@bps12 #2	0.35	6.20	1.21
GG@bps12 #3	0.32	6.10	1.24

Table S2. Wilcoxon rank-sum test of the rupture forces with ND and without ND for GG@bps7 and GG@bps12 (main text Fig.3A), respectively. P-values less than 0.05 indicate that this test rejects the null hypothesis at the 5% significance level.

X	Y	Alternative hypothesis	Statistic	P-value
GG@bps7+ND	GG@bps7	X>Y	2.2992	0.0107
GG@bps12+ND	GG@bps12	X>Y	2.7843	0.0027

Table S3. Wilcoxon rank-sum test of the dwell time with ND and without ND for GG@bps7, GG@bps12, and CG duplex (main text Fig. 4). P-value less than 0.05 indicates that this test rejects the null hypothesis at the 5% significance level.

X	Y	Alternative hypothesis	Statistic	P-value
GG@bps7+ND	GG@bps7	X>Y	8.6095	2.78e-18
GG@bps12+ND	GG@bps12	X>Y	17.8576	1.26e-71
CG duplex+ND	CG duplex	X>Y	-0.8035	0.7892
